# Periodic paralysis across the life course: age-related phenotype transition and sarcopenia overlap

**DOI:** 10.3389/fneur.2024.1507485

**Published:** 2024-12-23

**Authors:** Karen Suetterlin, Sinead Law, William David Arnold

**Affiliations:** ^1^AGE Research Group, NIHR Newcastle Biomedical Research Centre, Newcastle Upon Tyne Hospitals NHS Foundation Trust, Cumbria, Northumberland, Tyne and Wear NHS Foundation Trust and Faculty of Medical Sciences, Newcastle University, Newcastle upon Tyne, United Kingdom; ^2^The John Walton Muscular Dystrophy Research Centre, Institute of Genetic Medicine, Newcastle upon Tyne, United Kingdom; ^3^NextGen Precision Health, University of Missouri, Columbia, MO, United States; ^4^Department of Physical Medicine and Rehabilitation, University of Missouri, Columbia, MO, United States; ^5^Department of Medical Pharmacology and Physiology, University of Missouri, Columbia, MO, United States; ^6^Department of Neurology, University of Missouri, Columbia, MO, United States

**Keywords:** ageing, myopathy, sarcopenia, channelopathy, life course, mitochondria, skeletal muscle, periodic paralysis

## Abstract

In Periodic Paralysis (PP), a rare inherited condition caused by mutation in skeletal muscle ion channels, the phenotype changes with age, transitioning from the episodic attacks of weakness that give the condition its name, to a more degenerative phenotype of permanent progressive weakness and myopathy. This leads to disability and reduced quality of life. Neither the cause of this phenotype transition, nor why it occurs around the age of 40 is known. However, 40 is also the age of onset of ‘normal’ age-related physiological decline when we consider (a) muscle mass and strength (b) physical function at the world class level and (c) age-related mitochondrial dysfunction. Elevated Na^+^, mitochondrial dysfunction and sarcoplasmic Ca^2+^ leak via the skeletal muscle ryanodine receptor (RyR1) have been implicated in both periodic paralysis myopathy and skeletal muscle ageing. We suggest this combination may trigger a negative spiral ultimately leading to progressive muscle failure. Understanding the interaction between ageing physiology and disease phenotype will provide a window into the healthy ageing process but also help understand how, and why PP phenotype changes with age. Understanding the mechanism underlying PP phenotype-transition and its link with ageing physiology, not only has the potential to identify the first disease modifying therapies for PP, but also to identify novel and potentially tractable mechanisms that contribute to sarcopenia, the pathological loss of muscle mass and function with age.

## Introduction

The primary periodic paralyses are rare inherited conditions caused by skeletal muscle ion channel mutations. These disorders are broadly divided into Hyperkalaemic Periodic Paralysis (HyperPP), Hypokalaemic Periodic Paralysis (HypoPP) and Andersen Tawil Syndrome (ATS). HyperPP and HypoPP, as their names suggest, are associated with high or low serum potassium levels, respectively. HyperPP is caused by mutations in the skeletal muscle voltage-gated sodium gene (*SCN4A*). HypoPP, in Caucasian populations, is most commonly caused by mutations in the voltage-gated calcium channel gene (*CACNA1S* (HypoPP I)), whilst in Chinese populations HypoPP secondary to mutations in *SCN4A* (HypoPP II) is more common (1). Andersen Tawil Syndrome is caused by mutations in the inwardly rectifying potassium channel encoded by *KCNJ2* and, in contrast to Hyper and HypoPP, has extra-muscular manifestations of cardiac conduction abnormalities and dysmorphism.

**Figure 1:**
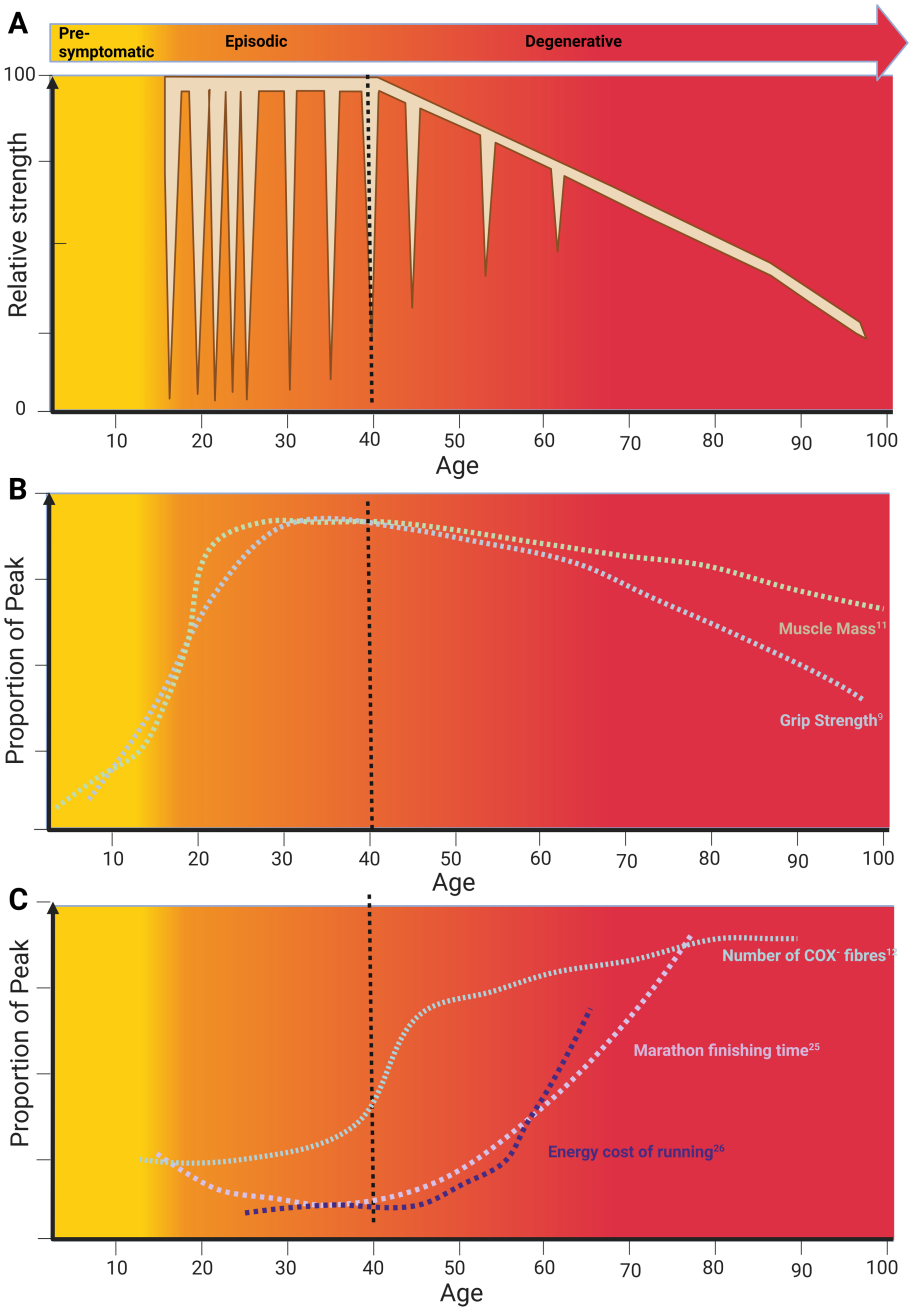
A life course approach to the effect of skeletal muscle ageing on periodic paralysis phenotype. **(A)** The life course of periodic paralysis Phenotype. **(B)** The life course of sarcopenia. **(C)** The life course of skeletal muscle function. Data plots from primary references were adjusted to match age scale on the *x*-axis and relative magnitude on the *y*-axis. Original data: Muscle mass (11); grip strength (9), Cytochrome C-Oxidase Negative (COX^−^) Fibres (12), Marathon finishing time (27), running economy (28). Created in BioRender, Suetterlin (2024) https://BioRender.com/q49h014.

Classically, the phenotype of PP is characterized by episodic bouts of weakness with normal or near-normal inter-ictal muscle function (2). However, a consistent, yet unexplained, feature is an age-related transition from episodic weakness to fixed progressive weakness with signs of muscle degeneration (Figure 1A) (3–5). The pathophysiology of episodic bouts of weakness in PP is well understood (6–8), but neither the mechanisms that underlie the progressive, fixed weakness, nor the trigger for phenotype transition are known. Furthermore, whilst there are effective treatments to help manage episodic weakness in PP, disease-modifying therapies to prevent progressive, fixed weakness are lacking.

Based on previous reports, the average age at which patients transition from experiencing episodic weakness to developing progressive, fixed weakness is around 40 years (3–5). Notably, this age in the general population coincides with the onset of age-related physiological decline (Figure 1) when we consider (1) muscle mass and function, (2) decline of physical function at the world class level and (3) age-related mitochondrial dysfunction. In this mini review, we review what is known about PP phenotype transition with age, explore potential mechanisms, and discuss links to skeletal muscle ageing and sarcopenia.

## Life course of periodic paralysis and skeletal muscle ageing

### Life course of skeletal muscle ageing

Life course studies have shown that skeletal muscle mass and strength increase in early life, plateau and then decline from middle into old age (9–11) (Figure 1B). These studies correlate well with the life course of peak motor performance which also exhibits a non-linear course (Figure 1C).

Non-linear ageing is also evident in clinical measures where the physiology-pathology continuum changes with age (Figure 1C). For example, in those aged under 40, signs of mitochondrial dysfunction in the form of Cytochrome C-Oxidase (COX) deficiency on muscle biopsy are pathological. However, for those over 40, up to 5 COX negative (COX^−^) fibres are considered as within normal limits (12) (Figure 1C). This non-linearity of ageing is also seen at the molecular level where two periods of substantial transcriptomic, proteomic and metabolomic dysregulation have been described. These two periods occurred at approximately 44 and 60 years of age (13) coinciding with the reported age of phenotype transition in periodic paralysis. At 44 years cardiovascular disease, lipid, and alcohol metabolism are the major pathways affected. This is striking as the incidence of cardiovascular disease increases exponentially from the age of 40 (14).

**Figure 2:**
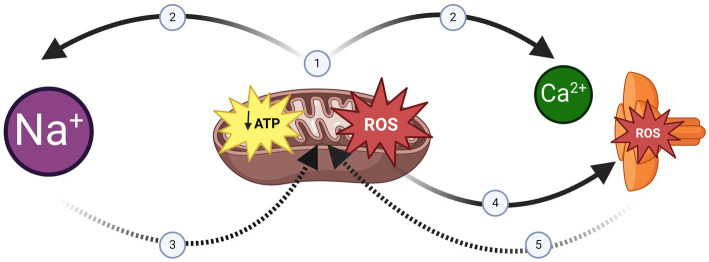
The negative spiral of mitochondrial dysfunction, sodium overload and RyR1 Leak. (1) Mitochondrial Dysfunction is a hallmark of ageing skeletal muscle (18) and Periodic Paralysis Myopathy (54). (2) Ionic homeostasis (e.g., Na^+^ extrusion/Ca^2+^ reuptake) is a major energy requirement of skeletal muscle (16). Impaired ATP synthesis would impact ionic homeostasis resulting in intracellular Na^+^ overload and Sarcoplasmic Reticulum Ca^2+^ depletion. (3) Na^+^ overload impairs mitochondrial dysfunction further (21). (4) Mitochondrial Free Radicals exacerbate Sarcoplasmic Reticulum Ca^2+^ depletion by inducing RyR1 Ca^2+^ leak (34). (5) Sarcoplasmic Reticulum Ca^2+^ depletion reduces the stimulus for Ca^2+^ induced ATP synthesis (30). Created in BioRender, Suetterlin (2024) https://BioRender.com/q49h014.

Skeletal muscle is one of the main regulators of lipid metabolism in the body (15). However, it also consumes nearly 80% of available glucose and regulates both basal metabolic rate and whole body energy expenditure (16, 17) so in addition to its role enabling movement, skeletal muscle is a key metabolic organ. Maintenance of the transmembrane ion gradients necessary for action potential initiation and propagation and skeletal muscle contraction and relaxation is a major energy cost. Maintaining the transmembrane Na^+^ gradient uses 7% of skeletal muscle ATP whilst Ca^2+^ reuptake into the sarcoplasmic reticulum accounts for 35% (16). This means that ionic homeostasis accounts for just under half of total skeletal muscle ATP use (16). Given that mitochondrial dysfunction is a key hallmark of skeletal muscle ageing (18), this suggests that ionic homeostasis may be compromised in older muscle. In support of this, skeletal muscle sodium content, as measured by Magnetic Resonance Spectroscopy, increases exponentially in men over age 40 (19). A phenomenon is also seen in wild-type mice where skeletal muscle sodium is elevated from middle age and continues to increase into old age (20). In cardiac tissue, Na^+^ overload reversibly inhibits mitochondrial ATP synthesis and increases free radical production (21). If the same were true for skeletal muscle, Na^+^ overload would itself exacerbate mitochondrial dysfunction further (Figure 2).

Additional evidence of a change in ionic homeostasis and skeletal muscle excitability with age comes from human Muscle Velocity Recovery Cycles (MVRCs). MVRCs are a specialised electromyography technique that use post-impulse change in conduction velocity, known as supernormality, as an indirect measure of skeletal muscle excitability and ion channel function *in vivo* (22) MVRCs are sensitive enough to detect the genetic ion channel dysfunction associated with periodic paralysis and can distinguish HyperPP from HypoPP (23). Life course studies of human MVRCs in periodic paralysis have not been performed. However, MVRCs do demonstrate change with age in healthy muscle membrane properties. These changes consist of an increase in muscle relative refractory period and a decrease in supernormality that would be most consistent with a relative depolarisation of the resting membrane potential and/or increased inactivation of the skeletal muscle voltage-gated sodium channel with age (24).

As well as altered ionic homeostasis, there is evidence that ionic homeostasis becomes more energy intensive with age. Whilst older subjects had similar ATP *synthesis* rates to younger subjects during continuous contraction, the ATP *cost* of intermittent contractions was significantly greater and correlated with dynamic single leg extensor peak power. This effect was attributed to the additional activity of Na^+/^K^+^ ATPase and Ca^2+^ ATPases required for intermittent, but not continuous contraction given the need to repeatedly depolarise-repolarise the membrane and contract-relax the muscle (25).

Other groups have also found an increased metabolic cost of muscle movement in older subjects. In fact, measures of energy cost show age-related deterioration with the point of inflection depending on the intensity of workload. For example, walking but not standing is associated with an increased ATP cost in older compared to younger subjects. Strikingly, this increase in energy expenditure/metre with age is an almost mirror image of the decline in gait speed with age (26). A similar phenomenon is apparent when comparing the timeline of change in marathon finishing time with age (27) and the increase in running energy cost with age (28) (Figure 1C). However, in this case, significant decline starts around the age of 40, approximately 20 years earlier.

In skeletal muscle, energy supply and demand are linked both structurally and functionally as mitochondria are tethered at the triad in the region of RyR1 (29) and Ca^2+^ efflux via RyR1, as well as triggering muscle contraction, triggers mitochondrial ATP production (30). This link between energy supply and demand is disrupted with increasing age as RyR1 channels become oxidised, nitrosylated and leaky (31, 32) resulting in reduced sarcoplasmic reticulum Ca^2+^ content (32, 33) and therefore Ca^2+^-induced contraction force. Increased reactive oxygen species production as a result of mitochondrial dysfunction increases RyR1 leak (34) and RyR1 leak itself exacerbates mitochondrial dysfunction (35) setting up a potential negative spiral (Figure 2).

As well as the intrinsic muscle factors described above, neural factors play a key role in age-related muscle failure (18, 36). There is a decline in motor unit number, a slowing of nerve conduction velocity and a reduction in motor unit firing rate (18). The reduction in motor unit firing rate has now been linked to clinically significant weakness in older adults (37). There is also selective atrophy of type II, fast-twitch fibres with age with atrophy of the motor nerves that innervate them. The interested reader is referred to relevant reviews (18, 36).

### Life course of periodic paralysis phenotype

Symptom onset in HypoPP II due to *CACNA1S* mutation is 10 ± 6 years whilst for HypoPP I due to *SCN4A* mutation it is 16 ± 5 years. This is striking as the allelic disorder HyperPP has an average age of onset of 2 ± 4 years (38–40), a full decade earlier than HypoPP. Given the disorders are caused by the same gene, this discrepancy cannot be accounted for by a change in ion channel expression alone. This suggests that a change in muscle physiology occurs around puberty and increases susceptibility to HypoPP attacks of weakness. The majority of ATS patients report symptom onset before age 10. However, presentation can be as late as the 6th decade (41).

The frequency and severity of both Hypo and HyperPP attacks change with age (38, 42, 43). This correlation is not as well established in ATS. Hyper PP attacks have been described as short and frequent in childhood becoming longer and more severe around puberty (38, 43). An increase in attack frequency and severity with peak during puberty and late adolescence is also described in HypoPP (40) (Figure 1A). The reason for this is not known but change in sex hormones and/or the number of ‘indiscretions’ triggering attacks (3) have been suggested. Biemond described ‘indiscretions’ associated with HypoPP as large carbohydrate loads, especially late at night, and rest after exercise whilst for HyperPP rest after exercise and cold were the main triggers.

In approximately half of the large HyperPP kindred originally described, attacks reduced in frequency and severity after age 50 or 60 to the point where attacks sometimes ceased (38). Similar findings were reported in a retrospective patient questionnaire (43) and in other case reports (42). In HypoPP, a reduction in attack frequency and severity starts to occur in adulthood and in many reports, episodic attacks cease around the age of 40 when fixed weakness develops (3–5, 44, 45).

However, it is not clear if and how the reduction of episodic weakness and the onset of fixed, degenerative weakness are causally related as patients with no antecedent history of paralytic attack can still develop permanent progressive weakness (45–48) and the age of onset of permanent progressive weakness can vary significantly. In some cases very young patients can be affected whilst in other cases older patients remain apparently unaffected (49). Perhaps somewhat surprisingly, young patients exhibit muscle fat infiltration – it was noted in 75% of those under 40 in a recent periodic paralysis MRI cohort study (49). However, although present under the age of 40, MRI muscle fat infiltration still significantly increased with age in all types of PP (49). This increase in muscle fat content correlates with increasing disability scores (49), a finding corroborated by 25% of the cohort requiring walking aids. Significant morbidity with increasing age was also noted in a very large HypoPP pedigree where all family members above the age of 70 were wheel chair bound (45).

Although muscle fat infiltration was seen in all types of PP, patients with *SCN4A* mutations were found to have the most severe fat infiltration, suggesting that the allelic disorders HyperPP and HypoPP II may be particularly susceptible to PP myopathy (49). Longitudinal MRI studies have demonstrated interval change in muscle fat infiltration over a 3 year follow up period in Hyper PP patients (50, 51).

As well as an increase in muscle fat infiltration, an increase in skeletal muscle sodium concentration as measured by ^23^Na^+^ Magnetic Resonance Spectroscopy techniques has been demonstrated in both HypoPP (52) and Hyper PP (50, 53). The ^23^Na^+^ signal increases reversibly during an induced attack of weakness (53). However, it is also elevated at baseline in HyperPP patients with fixed weakness where the magnitude of ^23^Na^+^ signal correlates with the severity of weakness (50). This Na^+^ elevation may itself exacerbate mitochondrial dysfunction (21) and thus further exacerbate ionic dyshomeostasis (Figure 2).

### Life course of periodic paralysis pathophysiology

The classical features of all forms of periodic paralysis myopathy are vacuoles and tubular aggregates (40, 41, 54, 55). Goldflam first reported the characteristic vacuoles of HypoPP myopathy in 1897. Since then some authors report that vacuoles are formed during the course of an induced attack (56, 57) whilst others state they are unrelated to paralytic episodes (58). There has also been a case report describing the presence of ‘target’ fibres in an affected gastrocnemius muscle that resembled core-like regions (59). An increase in sarcoplasmic glycogen, vacuolation of mitochondria and changes in the I band region of myofibrils are all consistent features seen in PP biopsy and considered secondary (54). A preferential and pathological reduction in size of type II fibres has also been noted (57). However, muscle structure may also be normal in patients with PP (40, 54) especially in young patients (54).

There are 4 published mouse models of periodic paralysis: one HypoPP I (60), one HypoPP II (61) and two HyperPP (6, 62). Life course studies have been performed in the Draggen mouse model of Hyper PP (63). Draggen mice have an autosomal dominant gain-of-function mutation Ile582Val in *SCN4A* that results in myotonia and episodic hind-limb immobility with a median onset age of 16 weeks in males and 25 weeks in females. Mirroring the reports of reduced attack severity with age in humans, old Draggen males were significantly more resistant to potassium-induced weakness than young or middle-aged ones (63). However, surprisingly, the same phenomenon was seen in old wild-type mice who were also significantly more resistant to potassium-induced weakness when compared to young or middle-aged wild-type males. This suggests the resistance to potassium-induced weakness is the effect of intrinsic muscle ageing rather than related to chronic ion channel dysfunction.

At around 60 weeks of age (human equivalent of ~40 years), Draggen mice develop fixed weakness with histological features of PP myopathy such as tubular aggregates (62). Tubular aggregates are also seen in aged male wild-type mice where their formation can be prevented by exercise (64). Draggen soleus also developed core-like regions with age (63) similar to what has been described in human PP (59) previously. Cores or core-like regions represent localised absence of mitochondrial or sarcoplasmic reticulum membrane and are classically associated with RyR1 myopathy. It was striking, therefore, that old Draggen mice also had significantly reduced caffeine contracture force with age (caffeine is an RyR1 agonist) and a decreased energy charge (ATP:ADP:AMP) following a 2 min protocol of increasing frequency of electrical muscle stimulation up to 30 Hz (63). This suggests an inability to maintain the energy demands of ionic homeostasis with age in Draggen mice.

The combination of core-like regions on muscle histology with reduced caffeine contracture force and decreased energy charge implicate acquired RyR1 dysfunction either causing, or secondary to, mitochondrial dysfunction. A strong connection between PP, RyR1 channels and mitochondria is suggested by the fact that RyR1 and mitochondrial mutations have been reported to exhibit an atypical periodic paralysis phenotype (65, 66) and coenzyme Q10, an electron carrier between respiratory chain enzymes, was effective in treating PP (67). Moreover, a transgenic mouse with RyR1 mutation had a HypoPP phenotype with core-like structures on histology and impaired ATP synthesis secondary to mitochondrial membrane depolarisation (35).

Thus, there is preclinical and clinical evidence implicating RyR1 and mitochondria in the episodic weakness phenotype of PP (35, 65–67). However, there is also new preclinical evidence linking RyR1 leak and mitochondrial dysfunction to the development of fixed progressive weakness with degenerative features (63). Therapies that target RyR1 leak have now completed phase I and are about to start phase II clinical trials (68). If RyR1 leak is confirmed to contribute to fixed, progressive weakness with degenerative features in humans with PP, RyR1 leak-targeted therapeutics could provide the first disease modifying treatments for PP. It follows that confirming the contribution of RyR1 leak to phenotype transition with age in patients with PP should be a priority.

## Parallels and insights

Firstly, it is important to highlight that a non-linear ageing trajectory is apparent in both periodic paralysis and age-related muscle failure. This emphasises the importance of a life course approach to the study of ageing as if we simply compare young and old, we risk mistaking consequence for effect.

Secondly, there are striking similarities in the time course of changes underlying healthy ageing and the phenotype change in periodic paralysis (Figure 1). This suggests similar mechanisms may be involved. Whilst, the energetic requirements of ionic homeostasis are increased with age (25), they are undoubtedly increased at baseline in periodic paralysis as evidenced by the effect of micromolar ouabain, a Na^+^K^+^ATPase blocker, on HypoPP II muscle where it caused membrane depolarisation and weakness whilst the same dose in wild-type muscle had no effect (61). This raises the possibility that the age-related impairment in ability to maintain ionic homeostasis tips periodic paralysis muscle over the edge. This could explain the increase in ^23^Na^+^ signal seen on MR imaging in HyperPP patients, the magnitude of which correlates with permanent weakness (50).

The evidence from MVRCs of a change in excitability with age may also help explain the transition from episodic to fixed weakness as, whether due to sodium channel inactivation or depolarisation of resting membrane potential, this could be sufficient to tip an already depolarised membrane past a critical threshold, resulting in fixed weakness. This excess depolarisation, combined with an inability to meet the ATP costs required to deal with it, may set up the negative spiral described in healthy ageing muscle of mitochondrial dysfunction increasing RyR1 leak which consequently impairs oxidative phosphorylation increasing RyR1 leak further (Figure 2). This negative spiral would be exacerbated by genetic ion channel dysfunction increasing energy requirements of ionic homeostasis. A proposal supported by the findings of impaired oxidative phosphorylation and acquired RyR1 leak in old Hyper PP mice (63).

What is less clear is how and why old muscle becomes resistant to potassium-induced weakness, and if and how this relates to the onset of a degenerative phenotype. One possibility is that depolarisation in the context of elevated intracellular Na^+^ triggers reverse mode of the Na^+^/Ca^2+^ exchanger. Reverse mode of the Na^+^/Ca^2+^ exchanger has been reported to protect against high-frequency fatigue in mouse soleus muscle (69). Thus, reverse mode of Na^+^/Ca^2+^ exchanger could alleviate potassium-induced weakness whilst exacerbating permanent progressive weakness with degenerative features by contributing to Ca^2+^ overload.

Future work needs to confirm the negative interaction between elevated Na^+^, mitochondrial dysfunction and RyR1 Ca^2+^ leak and clarify their role in both triggering and perpetuating the phenotype transition in PP. Understanding these mechanisms and their link with ageing physiology, has potential to identify the first disease modifying therapies for PP but also to identify potentially novel and tractable mechanisms that contribute to sarcopenia, the pathological loss of skeletal muscle mass and function with age.
